# Between borders and barriers: disability prevalence, pregnancy outcomes and maternal care access on the Thai-Myanmar border, a mixed methods study

**DOI:** 10.3389/fgwh.2026.1919504

**Published:** 2026-09-30

**Authors:** Qin van de Stadt, Taco Jan Prins, Suphak Nosten, Mupawjay Pimanpanarak, May Myo Thwin, Ko Ko Aung, May Mon Mon Theint, Aung Myat Min, Mary Ellen Gilder, Nay Win Tun, Dirk Essink, Chaisiri Angkurawaranon, Rose McGready

**Affiliations:** 1 Athena Institute, Vrije Universiteit Amsterdam, Amsterdam, Netherlands; 2 Shoklo Malaria Research Unit, Mahidol–Oxford Tropical Medicine Research Unit, Faculty of Tropical Medicine, Mahidol University, Mae Ramat, Thailand; 3 Department of Family Medicine, Faculty of Medicine, Chiang Mai University, Chiang Mai, Thailand; 4 Global Health and Chronic Conditions Research Group, Chiang Mai University, Chiang Mai, Thailand; 5 Department of Internal Medicine & Infectious Diseases, Amsterdam University Medical Centres, and Research Groups: APH, GH and AII&I, Amsterdam UMC, Amsterdam, Netherlands; 6 Nuffield Department of Medicine, Centre for Tropical Medicine and Global Health, University of Oxford, Oxford, United Kingdom

**Keywords:** community, disability, healthcare workers, low resource setting, maternal care perspectives, migration, pregnancy outcomes

## Abstract

**Introduction:**

Research on the intersection of displacement, disability, and pregnancy remains scarce globally. At the Thai–Myanmar border tens of thousands of displaced individuals including over 12,000 refugees with documented disabilities reside. The objective of this analysis is to assess the prevalence of pregnant People With Disabilities (PWD) among displaced populations and to explore perspectives on disability and barriers to accessing maternal care in this setting.

**Methods:**

This mixed-methods study included a retrospective analysis of 49,038 clinical records of refugees and migrants who gave birth or received antenatal care at clinics operated by the Shoklo Malaria Research Unit. Logistic regression was used to compare pregnancy outcomes between pregnant people with and without disabilities. The qualitative component included five in-depth interviews with migrant PWD and five focus group discussions with healthcare staff and migrant community health volunteers providing maternal care. Deductive and inductive thematic analyses were conducted to examine experiences of accessing, receiving, and providing care.

**Results:**

Documented prevalence of pregnant PWD was low at 0.62% (305/49,038; 95% CI 0.55–0.70). Women with disabilities reported barriers including stigma, transportation difficulties, financial constraints, and reliance on accompaniment to attend clinics. Those who accessed care described respectful communication and supportive interactions with staff. Healthcare providers expressed strong commitment to equitable care. Quantitative findings included higher antenatal care attendance and lower loss to follow-up among women with disabilities.

**Discussion:**

The low documented prevalence of disability may reflect underrepresentation or underreporting of pregnant PWD in maternal services or a healthy migrant effect. Quantitative results reported favourable ANC use and perinatal outcomes, which aligned with accounts of respectful communication, equal treatment, and participation in decision-making. Future research should use standard tools, disaggregate findings by disability type, severity, and ethnicity to uncover specific needs.

## 1 Introduction

People with disabilities (PWD) experience lower educational attainment, and higher rates of unemployment than people without disabilities. Factors that exacerbate vulnerabilities in maternal health include access to quality antenatal, intrapartum, and postnatal care (1). The inequities of PWD are often compounded in contexts of forced displacement. PWD are frequently left behind and or contend with systemic barriers restricting access to essential health services (2). Disability is therefore increasingly recognized as a critical determinant of health disparity, yet its intersection with displacement and maternal health, particularly in low resource, humanitarian settings, remains underexplored (1).

Myanmar, formerly known as Burma has endured persistent civil unrest and ethnic conflict since gaining independence in 1948, resulting in the displacement of nearly 7 million people (3, 4). Consequently, the Thai-Myanmar border has become a humanitarian setting where approximately 82,400 people have sought refugee, often working in low-wage informal sectors, without legal documentation (5–7). The healthcare system at the border can be described as liminal, meaning that it consists of “*a constellation of multiple, formal and informal health system arrangements at the local level that together provide health services around the border amidst recurring shocks, protracted crises, and conflict-induced movements of resources, funding, and people”* (8)*.* Displaced pregnant women at the Thai-Myanmar border face numerous barriers in accessing health such as fear of arrest or deportation, limits to financial resources and language constraints (7, 9, 10).

Among the displaced individuals along the border, many people have a registered disability, representing a particularly vulnerable group (5, 6). A disability can further increase barriers due to social stigma, inadequate clinical expertise, and unsuitable infrastructure (11, 12). Disability is therefore not only a health condition but also an important social determinant of health, influencing educational opportunities, employment, social participation, and access to healthcare. These intersecting disadvantages can increase the risk of delayed or inadequate maternal care (1, 13).

Research indicates that pregnant PWD have higher risks of inadequate prenatal care and complications, and unfavourable pregnancy outcomes such as low birth weight and preterm neonates compared to non-disabled pregnant women (14–17). It is important to recognize the needs of disabled migrants as their conditions can often be exacerbated as a direct result of delays in necessary care (13).

Research on the intersection of displacement, disability, and pregnancy remains scarce globally. Currently, no data exists on the prevalence of disability among displaced pregnant women in the Thai-Myanmar border area, nor on the specific barriers they face in accessing maternal care. This study aimed to: (1) identify the prevalence, types and characteristics of disabilities among pregnant displaced women in this region, (2) explore the experiences of disabled displaced women in accessing and receiving maternal healthcare, and (3) examine the perspectives of both community-based and clinical healthcare workers on the challenges of providing care to this population.

## 2 Methods

### 2.1 Study design

A mixed-methods design was used, integrating quantitative and qualitative approaches to examine both the prevalence of disability and the complex perspectives involved in accessing, receiving, and providing maternal health care. This methodological integration enhanced the study's validity and reliability. The study was conducted and reported in accordance with the Strengthening the Reporting of Observational Studies in Epidemiology (STROBE) guidelines for observational research (18). As this study employed a mixed-methods design, elements of the Consolidated Criteria for Reporting Qualitative Research (COREQ) were also considered to ensure transparency in the qualitative component (19).

### 2.2 Study context

The Shoklo Malaria Research Unit (SMRU) has provided antenatal and perinatal care to refugees (since 1986, ending in 2016) and undocumented migrant women (since 1997) on the Thailand-Myanmar border in Tak Province (20). Refugee women are those who attend for care at SMRU clinics based in Maela Refugee camp and migrants attend for care at SMRU migrant clinics, based south of Maela in Mae Ramat and Phob Phra districts. Most refugee and migrant women who attend the clinics of the SMRU have a Karen or Burmese ethnicity. The clinical staff (midwives, nurses, medics, sonographers, counsellors and lab technicians) of the SMRU consist predominantly of locally trained individuals from Burmese and Karen border communities, supported by qualified doctors form diverse backgrounds. Since 2021, SMRU has worked more closely with migrant community health volunteers (CHV) in Mae Ramat and Pho Phra subdistricts.

### 2.3 Population

All women with singleton pregnancies who gave birth and/or attended SMRU ANCs between 2009 and 2023 were included in the quantitative components. Pregnancies were excluded from the analysis of the pregnancy outcomes if the outcome or Estimated Gestational Age (EGA) was unknown, or if EGA at birth was <28 weeks. In the process of making this distinction, contextual factors such as living situation, working conditions and socio-cultural customs played a large role. For example, a goitre was not considered a disability due to its prevalence in the region and the limited functional impact it typically has. In contrast, clubbing of the fingers or toes was considered a disability, as it may impair mobility or dexterity and the deformity itself can lead to social exclusion. Disabilities were categorized as physical (e.g., mobility impairments and long-standing health conditions such as asthma and rheumatoid arthritis), sensory (e.g., deafness, blindness, partial sightedness), or psychological/cognitive (e.g., psychological disorders such as psychosis, mental health issues, for example depression or suicidal ideation or cognitive impairments). Long-standing chronic conditions were classified as physical disabilities when available clinical descriptions indicated substantial and functional limitations, considering the low resource setting with limited assistive devices, restricted long-term treatment and demanding working conditions. All cases with a possible disability were discussed among three researchers. Ambiguous cases were resolved through consensus.

### 2.4 Quantitative component

Anonymized medical records of women receiving maternal care from the SMRU with an outcome of pregnancy between 01-Jan-2009 till 31-Dec-2023 were retrospectively reviewed (*n* = 49,038). Throughout this fifteen-year period a single, electronic, purpose-built application for ANC and birth care at SMRU facilities was used by staff for data collection. It is inevitable that there would be staff changes during the timeframe of the study but the leading local staff has remained consistent in the clinics. The dataset for the purposes of this research was first accessed and extracted on 12-Mar-2025, updating and clarification was completed by 12-Apr-2025.

The first trimester of pregnancy was defined as <13 weeks of pregnancy. Preterm birth was defined as birth between 28- and <37-weeks' gestation. Small for gestational age (SGA) was defined as birth weight below the 10th percentile for gestational age using INTERBIO-21st Project (21). Gestational age at birth was estimated via ultrasound when available or, alternatively, by Dubowitz postnatal assessment if the first ultrasound during Antenatal Care (ANC) was performed later than an EGA of 28 weeks.

#### 2.4.1 Statistical analysis

Descriptive statistical analysis was performed using IBM SPSS Statistics for Windows, version 30. Frequencies and proportions were calculated, and chi-squared tests were conducted to compare characteristics between women with and without disabilities. Bivariate logistic regression analysis, presented as odds ratios with 95% confidence intervals, was used to assess associations between disability status and maternal outcomes. These outcomes included, adverse outcomes before 28 weeks of gestation (including miscarriage, abortion, ectopic pregnancy, and molar pregnancy), and the number of antenatal care visits received, using cut-offs of four and eight visits in accordance with previous and current WHO guidelines.

### 2.5 Qualitative component

Interview and focus group discussion (FGD) guidelines were developed using the accessibility framework of Levesque et al. (Supplementary Figure S1) and consulting community members with disability on the appropriateness and effectiveness of the questions (22). The first interview and FGD served as pilots. Five semi-structured interviews with migrant women with a disability and five focus group discussions with a total of 33 healthcare workers were conducted [3 Medical doctors, 7 Antenatal care staff members who provided the regular check-ups, 6 Midwives, and 17 CHV (CHV)]. A detailed description of the participants can be found in Supplementary Table S4. Eligible participants for the interviews were over 18 years old, had a disability, had received SMRU services in the past four years, and could speak Karen or Burmese. Convenience sampling was used as interview availability and recruitment was limited to a timeframe of four months, between March and June 2025. Four participants with a physical and one with a sensory disability, were identified in SMRU clinics using convenience and purposive sampling. Healthcare workers were included if they had provided care to disabled pregnant women in the past 12 months.

Interviews and FGDs were both audio-recorded, live-translated, and transcribed and analysed using a combined content and thematic approach in Atlas.ti version 25. A coding guide was created through both deductive (guided by sensitizing concepts) and inductive (emerging themes) analysis, proceeding through open, axial, and selective coding (Supplmentary Figure S2). Vertical and horizontal analyses captured individual narratives and cross-cutting patterns, with multiple researchers involved to ensure rigor and reliability.

### 2.6 Researcher reflexivity

The first author is a non-migrant researcher who does not identify as having a disability or prior experience of pregnancy. This positionality, shaped by academic training and socio-economic privilege, may have influenced the design, data interpretation, and interaction with participants. To reduce potential bias, reflexive strategies were applied throughout the study, including collaboration and discussion with local researchers experienced in pregnancy themselves and, or care of pregnant women but not identifying as PWD, incorporation of community-informed definitions of disability, and triangulation across data sources.

### 2.7 Ethical considerations

Ethical approval for retrospective analysis of medical records at the SMRU has been provided by the Oxford University Ethics Committee (OXTREC: 28–09), and the local community advisory board in Mae-Sot, Thailand (TCAB-4/1/2015). For additional qualitative data collection, the protocol for this study was submitted to the Research Ethical committee of the Faculty of Medicine, Chiang Mai University, with approval given FAM-2568-0047. In addition, local guidance from the Tak Community Advisory Board on how to perform this research in a valuable and respectful way was sought (reference code: TCAB202502). An information sheet and informed consent form were provided to all participants recruited for the interviews and FGDs. Written informed consent from the participant was obtained where possible; otherwise, an impartial literate witness signed on behalf of the participant. Personal identifiers were redacted or coded. For healthcare professionals, only general roles (e.g., healthcare provider) were disclosed for quotations to protect anonymity.

## 3 Results

### 3.1 Quantitative component

Between 2009 and 2023, a total of 49,038 pregnant women attended ANC and/or gave birth at the clinics of the SMRU, of which, 305 (0.62%) [95% CI 0.55-0.70] had a disability. Over time the proportion of pregnant PWD has slightly increased from 2009 to 2013 0.4 (79/19,682) [95% Confidence Interval (CI) 0.3–0.5], 2014–2018 0.8 (126/16,146) [95%CI 0.7–0.9] to 2019–2023 0.8 (100/13,210) [95% CI 0.6–0.9] There is a slight difference between migrant, 0.8 (103/12,770) [95% CI 0.7–1.0], and refugee, 0.6 (202/36,267) [95% CI 0.5–0.6], pregnant PWD. SMRU mostly served refugees before 2016 and migrants after 2016.

Pregnant PWD were significantly less likely to be lost to follow-up (17.7%, 54/305), compared to women without disability (22.5%, 10,982/48,747) (OR = 0.74, CI: 0.55–0.99, *p* < 0.05). Women who were lost to follow up were excluded from further analysis, as well as pregnant PWD with a pregnancy outcome before 28 weeks and multiple pregnancies (PWD 7.2%, 22/305, without disability 8.0%, 3,895/48,733). The inclusion and exclusion of the medical records for the analysis of the perinatal outcome is available in Figure 1 and information on missing data in Supplementary Table S3.

**Figure 1:**
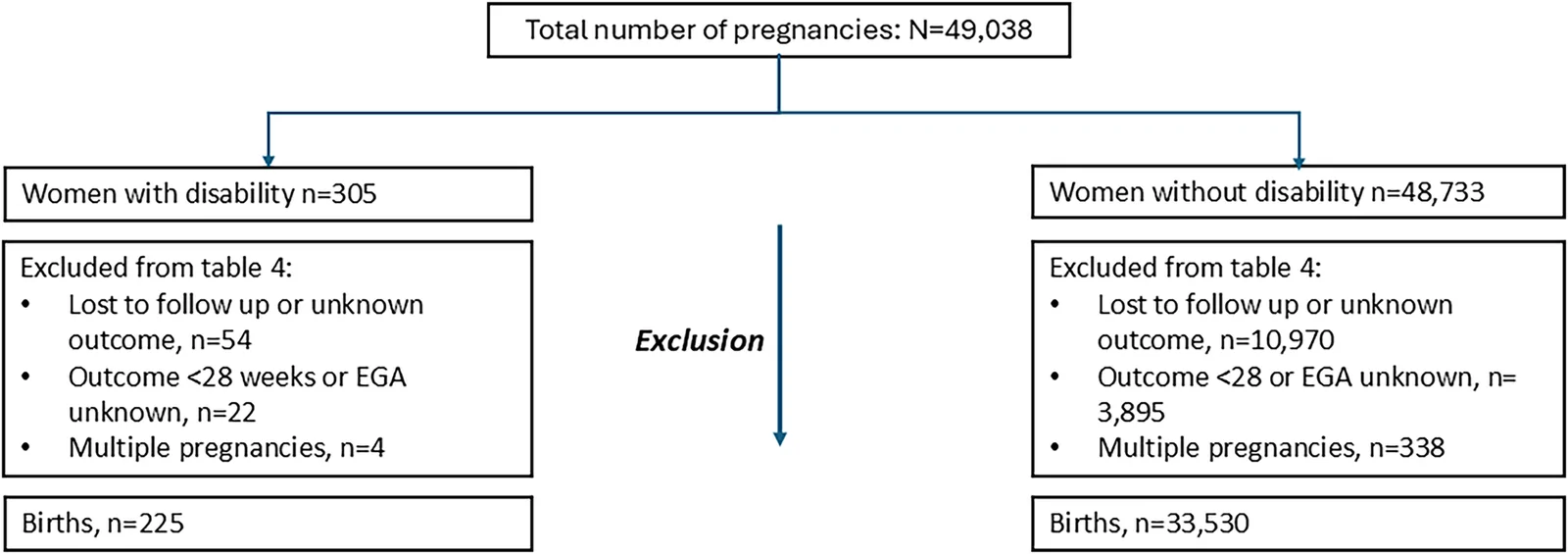
Visualization of the retrospective record review.

#### 3.1.1 Maternal characteristics

The age distribution of pregnant PWD was skewed towards older ages compared with women without disabilities. While most women in both groups were aged 20–34 years, this proportion was slightly lower among pregnant PWD (62.7%) compared to those without disabilities (67.9%) (Table 1). A higher proportion of pregnant PWD were aged ≥35 years (25.3%) than women without disabilities (13.9%) (*p* < 0.001). Literacy levels were lower in the group with disabilities: 48.8% of pregnant PWD reported being unable to read, compared to 33.9% among women without disabilities (*p* < 0.001). Pregnant PWD were more likely to initiate antenatal care (ANC) early, with 41.3% enrolling in the first trimester (<13 weeks), compared to 34.5% of women without disabilities (*p* = 0.015). Other maternal characteristics were not significantly different between groups.

**Table 1:** Characteristics of pregnant PWD receiving antenatal care with and without disability.

Characteristics 1st ANC	People with disability *N* = 225 Proportion (%)	Without disability *N* = 33,530 Proportion (%)
Age Mean (SD) [min.-max.]		
Age group	28.4 (7.7) [15-45]	26.1 (6.8) [13–53]
<20	27/225 (12.0)	6,086/ 33,529 (18.2)
20–34	141/225 (62.7)	22,779/33,529 (67.9)
35+	57/225 (25.3)	4,664/33,529 (13.9)
BMI (kg/m^2^) first trimester visit		
<18.5	19/93 (20.4)	2,093/11,578 (18.1)
18.5–24.9	59/93 (63.4)	7,937/11,578 (68.6)
25–29.9	13/93 (14.0)	1,242/11,578 (10.7)
>30	2/93 (2.2)	306/11,578 (2.6)
Gravida		
Primigravida	68/225 (30.2)	11,446/33,530 (34.1)
Multigravida	157/225 (69.8)	22,084/33,530 (65.9)
Parity		
Nulliparous	73/225 (32.4)	12,764/33,530 (38.1)
Multiparous	152/225 (67.6)	20,766/33,530 (61.9)
Can read		
Yes	110/215 (51.2)	19,830/29,980 (66.1)
No	105/215 (48.8)	10,150/29,980 (33.9)
Enrolled in 1st trimester <13 weeks		
Yes	93/225 (41.3)	11,581/33,530 (34.5)
No	132/225 (58.7)	21,949/33,530 (65.5)

#### 3.1.2 ANC trajectory and perinatal outcomes

Pregnant PWD were more likely to have a higher number of ANC visits (Table 2). Among pregnant PWD, 90.7% (204/225) attended at least four visits compared with 86.0% (28,849/33,530) among women without disability (OR = 1.58, CI: 1.03–2.55, *p* < 0.05), and 62.2% (140/225) pregnant PWD attended at least eight visits, compared with 54.4% (18,238/33,530) among women without disability (OR = 1.38, CI: 1.06–1.82, *p* < 0.05).

**Table 2:** Number of ANC visits, mode of birth and perinatal outcomes.

Outcome	People with disability *N*= 225 Proportion (%)	Without disability *N*= 33,530 Proportion (%)	Odds-ratio (95%-CI)
Nr. of ANC visits			
≥4	204/225 (90.7)	28,849/33,530 (86.0)	1.58 (1.03 −2.55)
≥8	140/225 (62.2)	18,238/33,530 (54.4)	1.38 (1.06—1.82)
Estimated Gestational Age at Birth (weeks): mean (sd) [min-max]	39 (2) [28–42]	39 (2) [28–45]	NA
Mode of Birth			
Vaginal Birth	197/225 (87.6)	31,316/33,530 (93.4)	NA
Caesarean Section	28/225 (12.4)	2,214/33,530 (6.6)	2.01 (1.32—2.94)
Skilled Birth Attendance	201/225 (89.3)	28,834/33,530 (86.0)	1.36 (0.91—2.14)
Preterm Labour			
28 to < 37 weeks	23/225 (10.2)	2,519/33,530 (7.5)	1.40 (0.89 −2.11)
≥ 37 weeks	202/225 (89.8)	31,011/33,530 (92.5)	NA
Small for gestational age	50/196 (25.5)	5,963/28,651 (20.8)	1.30 (0.94—1.79)
Apgar score 5 min. after birth			
<7	4/199 (2.0)	400/28,738 (1.4)	0.69 (0.29—2.24)
≥7	195/199 (98.0)	28,337/28,738 (98.6)	NA
Stillbirth	3/225 (1.3)	289/33,491 (0.9)	1.55 (0.38—4.10)

The mean estimated gestational age at birth was 39 weeks in both groups. A difference was found in mode of birth, 12.4% (28/225) of pregnant PWD gave birth through caesarean section, compared to 6.6% (2,214/33,530) of women without disability (OR = 2.01, CI: 1.32–2.94, *p* < 0.01).

Furthermore, pregnant PWD were more likely to give birth to a child that was small for gestational age (25.5%, 50/196) than women without disability (20.8%, 5,963/28,651, OR = 1.30, CI: 0.94–1.79).

#### 3.1.3 Differences between disability types

This study distinguishes three disability types. Among the 225 pregnant PWD, 127 (54.1%) had a physical disability, 30 (12.5%) pregnant women had a sensory impairment and 68 (32.1%) had a psychological/cognitive disability.

When disaggregating by disability type, the analysis of women with physical disabilities largely mirrors the broader comparison between women with and without disability (Supplementary Table S1, S2). Among women with sensory disability, a statistically significant difference was observed in age distributions. Furthermore, small sample sizes limited the statistical power to detect significant differences in the sensory and psychological/cognitive disability groups.

### 3.2 Qualitative component

Five interviews were held with pregnant PWD and five FGD's were conducted including 33 participants (Supplementary Table S3). Three main themes emerged: recognizing and reporting disability, accessing maternal care, and receiving and providing maternal care.

#### 3.2.1 “Well this is normal for her”: recognizing and reporting disability

Disability was commonly understood as a condition that limits daily functioning: “*If someone perceives that they are not disabled because they are able to solve all the problems of their daily life, or in this specific context, you don't want to put a label on them” (-FGD1 Doctors).* The ANC staff explains: “*[If] they have [a] normal leg, normal arm, they can walk, they can do things. That means they are not disabled” (-FGD2 ANC)*.

While every patient has a system based physical examination for cardiovascular, respiratory and neurological functioning, pregnant PWD might be missed or not labelled as such as more in-depth approaches are not standardised. Women with cognitive disabilities were particularly difficult to identify, as they often appeared to understand instructions but later proved unable to follow them. Sometimes, the staff, who often have a more closely related background to the migrant population, don't report a disability in the patient file and it is only recognised later by the medical team after multiple visits, that a woman had a disability. “*The patient comes in and she has an obvious limp. You know these obvious things that are, we could say abnormalities, that are good to document but they don't get reported because to the staff, it's like well this is normal for her so we just accept this as normal” (-FGD1 Doctors).* The women themselves also don't declare their disability, one of the doctors' reasons that this is because of the limited options available for pregnant PWD: “*There's such a huge difference in services for disabled people in this setting, since they don't exist here, then why declare your disability? Because it's not going to win you anything and it's not going to get you anywhere’ (-FGD1 Doctors)*.

The current differing ways of recognizing and reporting disability may contribute to an underestimation of disability prevalence. The doctors therefore expressed the need for a systematic and formal way of assessing disability to tailor the care to be timely and effective for the needs of the patient.

#### 3.2.2 “I try to encourage myself to go to the clinic”: accessing maternal care

The data suggests that pregnant PWD are less able to access maternal care services due to various barriers. In the community, disability is considered to be one of many difficulties and therefore difficult to support: “*Everyone is a migrant worker, everyone is a daily-wage worker, we cannot take care every time. If someone [from the community] has sympathy, they support a little bit, but they [PWD] need to survive themselves”(-FGD4 CHV's).* In turn, pregnant PWD themselves are hesitant to ask for help: “*I don't know how to ask for help, I can't get any help because everyone has to work” (-I4, physical disability)*. PWD were perceived to experience greater food insecurity and barriers to employment, as most available jobs involved physically demanding labour. CHVs attempted to bridge these gaps but noted resource constraints*: 'Sometimes we can help, but it is different because they have a disability; if you give 100 Baht to a healthy person, you need 200 Baht for a person with disability. More financial support [is needed] for a person with disability' (-FGD4 CHV's)*.

Normative barriers included exclusion from social gatherings, ridicule, and risks of abuse leading to safety concerns for PWD: “*No one cares about their [PWD] lives, they don't have any protection it is not safe, the neighbour can come for rape” (-FGD2 ANC).* In general, cognitive disabilities are associated with greater stigma in comparison to other disability types as community members are often afraid of people with cognitive disabilities.

Pregnancy among PWD was perceived in contradictory ways. The CHV's considered having children as a human right, yet several interviewees reported negative experiences such as gossip, staring or pressure to terminate pregnancies, I5 explains: “*I try to tell myself that this [disability] is not my fault and encourage myself to go to the clinic. Other women are staring at me or talking behind my back which often makes me feel down” (-I5, physical disability)*.

A lack of suitable public transportation posed additional obstacles as pregnant PWD depended on relatives, often husbands, to accompany them. This often resulted in lost income, I1 explains: ‘'*Our situation is very difficult, we only have each other, so if I need to come to the clinic, my husband cannot work because he has to accompany me, which further worsens our financial situation” (-I1, sensory disability)*. These structural and normative barriers compounded to limited access to care.

#### 3.2.3 “*I get treated the same as others”:* receiving and providing maternal care

All pregnant PWD interviewed expressed satisfaction with the maternal care they received. I5 states: “*I came here two times already, when I am sick I get treatment and our [her and the healthcare worker's] relationship is really good. I get treated the same as others’ (-I5, physical disability).* Information was given in ways adapted to literacy and cultural differences, and women felt comfortable asking questions.

The healthcare providers demonstrated a willingness to take the (extra) resources and time needed to support pregnant PWD: “*We can spend more money, we are willing to do more, take care more” (-FGD4 Outreach)*. They reported adapting communication styles, making home observations, and referring complicated cases to hospitals.

However, challenges in caring for pregnant PWD with especially cognitive disability remained: *“During the labour pain, we encourage them to walk or drink water. They said “ok” and then they don't do it, they don't listen” (-FGD1 Doctors).* Pregnant PWD with cognitive disability are often afraid and it takes time and a calm and patient communication style to build trust “*When I saw her for the first time, when I would give an injection, she was afraid and not willing to accept it. But if you talk soft, don't shout, then she is okay” (-FGD3 Midwives).* Sometimes, the staff feels uncomfortable to take care of a person with a cognitive disability, as they sometimes become aggressive when they are in pain or afraid.

Relatives, especially husbands, were commonly seen as essential to care. It is important that the relative is aware of their import role in the care process and takes responsibility: “*The husband needs to help the whole time, he is the one the patient trusts. If he wants to go to the bathroom or have a cigarette, he cannot do, we will call him back” (-FGD3 Midwives)*. Extra counselling to the husband/relative in what the disability entails and how they need to support the woman, can help in creating this awareness.

No major concerns were raised about the clinic's physical infrastructure, even though one interviewee, paralyzed by spinal tuberculosis, needed to be physically carried to the toilet by her husband. Some adjustments were proposed to make the clinic more accessible, but providers questioned the value in a low-resource setting with few patients with disability: “*But we would use it [the wheelchair] so rarely. It's sort of like is it worth the investment? Would it get rusty? Wheelchairs are reasonably expensive” (-FGD1 doctors).* The data suggests that at this point, such improvements are nice to have rather than need to have; indeed, the clinic donated the wheelchair to the said patient.

## 4 Discussion

As evidenced by the results, we identify two main concluding narratives.

First, the amount of disability reported in the medical record database was low, with only 0.62% of the pregnant women having a disability. Studies with a similar sampling strategy and context remain limited and approaches to measuring disability vary greatly, making it difficult to compare prevalence rates (1). Nevertheless, in 2023 the disability rate among women (not pregnant women) in lower income countries was estimated as 22.1%, and 20.6% in Myanmar in the same year. This further underscores the low prevalence rate (23). There are a range of reasons for the low prevalence rate: underrepresented, underreported or left behind. Underrepresented could arise from the structural and normative barriers to accessing care such as negative social attitudes toward pregnant PWD, the difficulty in finding time off work both financially and in terms of employer support, the lack of appropriate transportation systems and the need for accompaniment to the clinic. The findings resonate with previous literature (9, 11, 12, 24, 25). The fact that PWD may not reach the border may contribute to the low prevalence, as the literature describes that PWD are often not able to flee their place of origin (26). The lack of difference in prevalence rate between refugees and migrants supports this hypothesis, as both populations move cross-border. Additionally, only the migrant women who get pregnant come to the clinic, therefore this research excludes the ones without pregnancy. It is however important to note that PWD are sexually active, get pregnant and have successfully become mothers (27). Furthermore, PWD have an increased risk of becoming pregnant involuntarily as they are at heightened risk of sexual abuse (1). It is highly likely that a substantial proportion of pregnant PWD are unable to access maternal health care and therefore remain largely absent from both service provision and research data. Furthermore underreporting is more likely to occur when there is no possibility of access to specialised disability care (28). Additionally, disability is likely underreported in the clinical records due to the absence of a systematic approach to identification and documentation. As highlighted in the qualitative findings, disabilities, particularly less visible or cognitive disabilities, may go unrecognized or unrecorded, resulting in an underestimation of the true prevalence of disability in this population.

Second, pregnant PWD who reached care reported high satisfaction: once pregnant PWD accessed services, they reported receiving equitable and attentive care. Quantitative outcomes such as higher ANC attendance and lower loss to follow-up support this conclusion. Earlier studies report similar trends in ANC utilisation in both high- and low-income countries (24, 29, 30). The results contrast with those of a recent scoping review including 57 articles mostly conducted in European and North American countries reporting on racialized migrant women's experience of discrimination when using maternal healthcare service. “*Accessibility problems, non-utilization of interpreters, and untimely and delayed care to disrespect, abuse, and differential care”* were identified; in discordance with statements such as “get treated the same as others” (3.3.2) reported here (31). Other studies also noted instances of provider mistreatment during delivery (24, 25, 29). The difference in findings might be caused by the fact that the health care providers in this study have a similar background (marginalised, migrant) and language (Burmese or Karen) background to the study population. This is important as cultural differences can unintentionally lead to cultural insensitivities (32). A different research lens would be required to determine if non-discrimination extends to care if refugee and migrant women receive care within the Thailand medical system.

Migrant women may be hesitant to be critical due to the prevailing strong cultural norm of politeness and consideration of others in the Burmese culture (33). Alternatively, they may fear that being negative could jeopardize their already constrained access to services (34). The positive findings might be perceived as contradictory considering the low documented prevalence rate, but they are likely to reflect different stages of access to care. The inequities occur before pregnant PWD enter the healthcare system and prevent them from accessing maternal care services altogether. The ones who overcome barriers receive satisfactory care. The experiences captured in this study reflect those of women who were able to navigate the barriers rather than the broader population of pregnant PWD along the Thai-Myanmar border.

The interviewees did not mention any challenges to accessibility due to the infrastructure in the clinic. This contrasts with other papers, where infrastructural challenges are highlighted (12, 13, 28). This may be explained by the fact that living conditions at home also offer limited adjustments to the needs of pregnant PWD. Additionally, the intense physical and mental challenges of migrating without authorization may select for highly resilient individuals who therefore will not express dissatisfaction easily. Most of the health clinics are built as single level structures.

Pregnant PWD need to be referred to a hospital for advanced care more often than women without disability. This is similar to the paper of Signore et al., stating that that complications may be more common among women with (physical) disability (35). In this study, the mode of birth was more often a caesarean section in pregnant PWD compared to women without disability, particularly those with physical disability, an observation supported by other studies as well (28, 36–40). In terms of perinatal outcomes, pregnant PWD were more likely to give birth to a baby that was SGA, compared to women without disability. A result comparable to previous studies (23, 30). However, unlike other studies, this research did not observe significantly higher rates of preterm birth.

### 4.1 Strengths and limitations

This study is, to our knowledge, the first to examine the intersection of disability, displacement, and pregnancy along the Thai–Myanmar border. The mixed-methods design enabled triangulation and provided a richer understanding of the factors involved (38). The conceptualisation of disability informed by local healthcare workers and community members, ensured cultural and contextual relevance. Several limitations must be acknowledged. The quantitative results relied on retrospective electronic medical records without standardized disability screening tools such as the Washington Group Questions, limiting the comparability of the findings to other disability studies (39, 40). However, given the low prevalence observed, under identification is likely to have had a greater effect on the results than over-classification (39). In addition, the medical records may not capture the participants' own perceptions of their condition. More details may have been written in the hand-held paper record that stays with the patient. It may well represent an under-estimate or reflect a certain level of health and mobility as a baseline for being an undocumented mobile and marginalised pregnant migrant or refugee. Second, the study did not include those unable to reach care which introduces a potential selection bias. Some categories were underrepresented or absent in the data collection, for instance only one woman with a sensory disability and no women with cognitive or psychological disability were interviewed. Although recurring patterns emerged across interviews and were corroborated by perspectives from healthcare providers and community health volunteers, thematic saturation could only be achieved for a relatively narrow range of disability experiences. The themes identified are likely to reflect the experiences of women with physical disabilities more strongly than those of other disability groups who may face additional barriers not fully captured in the study.

Due to the relatively small numbers, the quantitative analysis relied on bivariate logistic regression and did not adjust for potential confounding factors despite observed differences in population characteristics between women with and without disability. Consequently, the reported associations should be interpreted with caution and might not reflect independent effects of disability. Additionally, small sample sizes necessitated combining categories (e.g., psychological and cognitive disability) which may obscure meaningful distinctions. Cultural limitations must also be considered, such as the lack of distinction between Burmese and Karen participants, language barriers and cultural norms introducing social desirability bias. However, when criticism or suggestions were voiced, they may be especially meaningful given these cultural constraints (33, 40). Finally, while the study focused on SMRU, which has provided free maternal care services, over many years along 200 kilometers of the border in refugees and migrants, the generalizability of the findings to other similar settings needs confirmation.

### 4.2 Implications and recommendations

Clinical practices need to be reviewed to ensure that disabilities are accurately identified and sufficiently documented in medical records. The 0.6% prevalence rate of disability observed in this study, suggests a need for improved recognition and reporting. Clinical audits represent a cost-effective method for improving disability identification, providing locally relevant insights and enhancing the quality of care. Strengthening the skills of clinical and community based health staff towards a set of standard disability questions as developed by the Washington Group would be helpful (40). This tool is a useful starting point as it has been used in humanitarian settings and lends itself to short surveys which could be considered as an addition to antenatal care screening beyond the current physical and obstetric examination (39). A blinded and comparative study of current practice versus the Washington Group (short set on functioning) would help confirm the prevalence and shed light on the necessity and benefits of standardized screening.

Further research is needed to develop contextually appropriate approaches for disability screening and recording in maternal healthcare services. Additionally, examining social attitudes toward (pregnancy and) disability could inform community-based interventions enhancing inclusion and acceptance of PWD, thereby improving accessibility to maternal services. A prospective community based study is needed to more accurately assess the prevalence of disability in this population. This would help capture individuals who are currently unable to access maternal services and whose voices were not incorporated in this study. Further research is needed across a broader range of disability types, ideally incorporating distinctions by severity (mild, moderate, severe), and by ethnicity (e.g., Burmese *versu*s Karen) to add more nuance and to uncover culturally specific experiences. Comparative studies involving different healthcare providers in the region could improve the generalizability of findings. In the absence of social support systems, CHV's are often essential in assisting pregnant PWD. CHV's emphasised the need for assistive devices and financial resources to support basic needs and transport to the clinic. When accessibility to maternal care services improves, and more pregnant PWD are able to reach the clinic, the need for infrastructural adjustments, a structured disability identification and documentation system, a consistent care team and training to enhance skills become more prominent.

## 5 Conclusion

This study identified only 0.62% of pregnant PWD among displaced pregnant individuals, representing those who were able to navigate the structural and normative barriers described in the qualitative findings. Encouraging medical staff to report all disabilities irrespective of severity is required. Quantitative results reported favourable ANC use and perinatal outcomes, which aligned with the women's accounts of respectful communication, equal treatment, and participation in decision-making. Healthcare providers expressed openness to adapting care and dedicating additional time and resources to support pregnant PWD.

## Data Availability

The datasets presented in this article are not readily available because data cannot be shared publicly because this is a population of undocumented refugees and migrants and we do not have their permission to share their data. Data are available from the Mahidol-Oxford Research Unit Institutional data access committee upon reasonable request from researchers who meet the criteria for access to confidential data. Enquiries should be directed through the Bioethics and Engagement team coordinates Mahidol Oxford Research Unit's Data Access Committee (datasharing@tropmedres.ac). Requests to access the datasets should be directed to datasharing@tropmedres.ac.
