# Supplementary material for: Between borders and barriers: disability prevalence, pregnancy outcomes and maternal care access on the Thai-Myanmar border, a mixed methods study
Source: Front Glob Womens Health. 2026 Sep 30;7:1919504. doi: 10.3389/fgwh.2026.1919504 (PMC13661930; doi:10.3389/fgwh.2026.1919504)
Supplement: Supplementary file 1 [file Datasheet1.docx]

Supplementary Material

**S1 Table. Characteristics of pregnant people with different types of disabilities**

|  | People with disability  N= 225  Proportion (%) | | | Without disability  N= 33,530  Proportion (%) |
| --- | --- | --- | --- | --- |
| Characteristics | **Physical**  **N= 127** | **Sensory**  **N= 30** | **Psychological/**  **Cognitive**  **N= 68** |  |
| Age  Mean (SD)[min– max]  <20  20-34  35+ | 28.8 (7.8) [16-45]  12/127 (9.4)  81/127 (63.8)  34/127 (26.8) | 28.4 (8.5) [16-45]  6/30 (20.0)  16/30 (53.3)  8/30 (26.7) | 27.6 (7.2) [15-41]  9/68 (13.2)  9/68 (64.7)  15/68 (22.1) | 26.1 (6.8) [13-53]  6,086/ 33,529 (18.2)  22,779/33,529 (67.9)  4,664/33,529 (13.9) |
| BMI first trimester  <18.5  18.5 – 24.9  25 – 29.9  >30 | 10/50 (20.0)  30/50 (60.0)  9/50 (18.0)  1/50 (2.0) | 0/9 (0)  7/9 (77.8)  1/9 (11.1)  1/9 (11.1) | 9/34 (26.5)  22/34 (64.7)  3/34 (8.8)  0/0 (0) | 2,093/11,578 (18.1)  7,937/11,578 (68,6)  1,242/11,578 (10,7)  306/11,578 (2.6) |
| Parity  Nulliparous  Multiparous | 47/127 (37.0)  80/127 (63.0) | 12/30 (40.0)  18/30 (60.0) | 14/68 (20.6)  14.68 (79.4) | 12,764/33,530 (38.1)  20,766/33,530 (63.0) |
| Gravida  Primigravida  Multigravida | 42/127 (33.1)  85/127 (66.9) | 12/30 (40.0)  18/30 (60.0) | 14/68 (20.6)  54/68 (79.4) | 11,446/33,530 (34.1)  22,084/33,350 (65.9) |
| Can read  Yes  No | 65/120 (54.2)  55/120 (45.8) | 12/27 (55.6)  15/27 (44.4) | 33/68 (48.5)  35/68 (51.5) | 19,830/29,980 (66.1)  10,150/29,980 (33.9) |
| Enrolled in 1^st^ trimester <13  Yes  No | 50/127 (39.4)  77/127 (60.6) | 12/27 (44.4)  25/27 (55.6) | 34/68 (50.0)  34/68 (50.0) | 11,581/33,530 (34.5)  21,949/33,530 (65.5) |

*p<0.05 **p<0.01

**S2 Table. Number of ANC visits, mode of birth and perinatal outcomes**

|  | People with disability  N= 225  Proportion (%) | | | Without disability  N= 33,530  Proportion (%) |
| --- | --- | --- | --- | --- |
| Outcome | **Physical**  **N=127**  **Proportion (%)**  **OR (95% CI)** | **Sensory**  **N=30**  **Proportion (%)**  **OR (95% CI)** | **Psychological/**  **Cognitive**  **N=68**  **Proportion (%)**  **OR (95% CI)** |  |
| Nr. Of ANC visits  >4  >8 | 116/127 (91.3)  1.71 (0.97 – 3.37)  81/127 (63.8)  1.48 (1.03 – 2.14)* | 26/30 (86.7)  1.05 (0.41 – 3.58)  17/30 (56.7)  1.10 (0.53 – 2.30) | 62/68 (91.2)  1.68 (0.79 – 4.34  42/68 (61.8)  1.35 (0.84 – 2.24) | 28,849/33,530 (86.0)  NA  18,238/33,530 (54.4)  NA |
| Estimated Gestational Age at birth (weeks) (SD); mean [min-max] | 39 (2) [28 – 42] | 39 (2) [35 – 42] | 39 (2) [29 – 42] | 39 (2) [28-45] |
| Mode of birth  Vaginal Birth  Caesarean Section | 109/127 (85.8)  NA  18/127 (14.2)  2.34 (1.37 – 3.75)** | 26/30 (86.7)  NA  4/30 (13.3)  2.18 (0.64 – 5.59) | 62/68 (91.2)  NA  6/68 (8.8)  1.37 (0.53 – 2.92) | 31,316/33,530 (93.4)  NA  2,214/33,530 (6.6)  NA |
| Skilled Birth Attendance | 116/127 (91.3)  1.72 (0.97 – 3.38) | 24/30 (80.0)  0.65 (0.28 – 1.76) | 61/68 (89.7)  1.42 (0.70 – 3.41) | 28,834/33,530 (86.0)  NA |
| Preterm Labour  28 to < 37 weeks  ≥ 37 weeks | 11/127 (8.7)  1.16 (0.59 -2.07)  116/127 (91.3)  NA | 4/30 (13.3)  1.89 (0.56 – 4.87)  26/30 (86.7)  NA | 8/68 (11.8)  1.64 (0.72 – 3.24)  60/68 (88.2)  NA | 2,519/33,530 (7.5)  NA  31,011/33,530 (92.5)  NA |
| Small for gestational age | 35/114 (30.7)  1.69 (1.12 – 2.49* | 5/25 (20.0)  0.95 (0.32 – 3.35) | 10/57 (17.5)  0.81 (0.39 – 1.53) | 5,963/28,651 (20.8)  NA |
| Apgar score 5 min. after birth  <7  ≥7 | 2/111 (1.8)  0.77 (0.24 – 4.67)  109/111 (98.2)  NA | 1/26 (3.8)  0.35 (0.07 – 6.31)  25/26 (96.2)  NA | 1/62 (1.6)  0.86(0.19 – 15.23)  61/62 (98.4)  NA | 400/28,738 (1.4)  NA  28,338/28,738 (98.6)  NA |
| Stillbirth | 2/127 (1.6)  1.84 (0.30 – 5.81) | 0/30 (0)  NA*** | 1/68 (1.5)  1.71 (0.10 – 7.78) | 289/33,491 (0.9)  NA |
|  |  |  |  |  |

*** Among people with sensory disabilities, 0% had a stillbirth compared to 0.9% in the non-disabled group. The perfect separation prevented logistic regression modelling.

|  | People with disability  N= 225  Proportion (%) | | | Without disability  N= 33,530  Proportion (%) |
| --- | --- | --- | --- | --- |
| Characteristics | **Physical**  **N= 127** | **Sensory**  **N= 30** | **Psychological/**  **Cognitive**  **N= 68** |  |
| Age | 0 (0) | 0 (0) | 0 (0) | 1 (0) |
| BMI first trimester | 77 (60) | 21 (0.7) | 34 (50) | 21,952 (65.5) |
| Parity | 0 (0) | 0 (0) | 0 (0) | 0 (0) |
| Gravida | 0 (0) | 0 (0) | 0 (0) | 0 (0) |
| Can read | 7 (5.5) | 3 (1.3) | 0 (0) | 3,550 (10.6) |
| Enrolled in 1^st^ trimester <13 | 0 (0) | 3 (1.3) | 0 (0) | 0 (0) |
| Nr. Of ANC visits | 0 (0) | 0 (0) | 0 (0) | 0 (0) |
| Estimated Gestational Age at birth | 0 (0) | 0 (0) | 0 (0) | 0 (0) |
| Mode of birth | 0 (0) | 0 (0) | 0 (0) | 0 (0) |
| Skilled Birth Attendance | 0 (0) | 0 (0) | 0 (0) | 0 (0) |
| Preterm Labour | 0 (0) | 0 (0) | 0 (0) | 0 (0) |
| Small for gestational age | 13 (10.2) | 200 (88.9) | 11 (16.2) | 4,696 (14.6) |
| Apgar score 5 min. after birth | 16 (12.6) | 4 (13.3) | 6 (8.8) | 4,792 (14.3) |

**S3 Table. Missing data**

**S4 Table. Description of either disability and pregnancy characteristics or role in providing maternal care of participants**

| Mode of Data Collection | Description of disability and pregnancy characteristics or role in maternal care |
| --- | --- |
| Interview 1 | Visual impairment, primiparous, accompanied by husband |
| Interview 2 | Mobility impairment, paralysed from the waist down due to spinal tuberculosis, primiparous, accompanied by husband |
| Interview 3 | Mobility impairment, incorrectly healed broken arm due to domestic violence, termination of pregnancy, multiparous, accompanied by a friend |
| Interview 4 | Mobility impairment, deformity of the hand due to domestic violence, multiparous |
| Interview 5 | Mobility impairment, lower leg amputation due to mine explosion, primiparous, accompanied by relative |
| FGD 1 | 3 Medical doctors |
| FGD 2 | 7 Antenatal care providers |
| FGD 3 | 6 Midwives |
| FGD 4 | 8 Community health volunteers |
| FGD 5 | 9 Community health volunteers |

**S1 Fig. Conceptual framework for healthcare access (Levesque et al., 2013)**

**S2 Fig. The coding guide used for qualitative data analysis emerging inductively/deductively from the data.**
